# Innovative use of intact seeds of *Mucuna monosperma* Wight for improved yield of L-DOPA

**DOI:** 10.1007/s13659-011-0051-3

**Published:** 2012-02-07

**Authors:** Shrirang Inamdar, Swati Joshi, Jyoti Jadhav, Vishwas Bapat

**Affiliations:** Department of Biotechnology, Shivaji University, Kolhapur, 416004 India

**Keywords:** 3-(3,4-dihydroxyphenyl)-L-alanine, elicitors, intact seeds, *Mucuna monosperma*, Parkinson’s disease, tyrosinase

## Abstract

The drug L-DOPA has been widely used against Parkinson’s disease and is extracted from plants. Due to the increasing demand of this drug, new plant sources need to be discovered in addition to the existing sources. The paper embodies results on *Mucuna monosperma*, which can be a promising candidate for L-DOPA. The seed powder of this plant contains 5.48% of (dry weight) the drug and when the seeds were soaked in distilled water, content was increased to 6.58%. Different elicitors when added, enhanced the drug level in seed up to 11.8%. The possible rationale behind this increase was confirmed by increase in tyrosinase activity in the seeds. Presence of L-DOPA was confirmed using various analytical techniques as HPLC, HPTLC and NMR. The work demonstrates a potential candidate plant as a source for L-DOPA when a novel method was adopted as described here.

**Electronic Supplementary Material:**

Supplementary material is available for this article at 10.1007/s13659-011-0051-3 and is accessible for authorized users.

## Introduction

Parkinson's disease (PD), a degenerative disease of the nervous system and is characterized by muscular rigidity, difficulty with balance and walking, depression and dementia.[1] The prevalence is variable, ranging from as low as 31/100,000 population in Libya to 300/100,000 population from Canada.[2] L-DOPA (3-(3,4-dihydroxyphenyl)-L-alanine), a dopamine precursor, either alone or in combination with an aromatic amino acid decarboxylase inhibitor is the most effective drug for the treatment of PD, since dopamine fails to pass through the blood brain barrier. Use of biological sources for production of L-DOPA, is always desirable and advantageous because the chemical synthesis results in racemic DL-mixture, which is inactive and further separation of enantiomerically pure L-DOPA from this mixture, is very difficult and cumbersome. In addition, D-DOPA interferes with the activity of DOPA decarboxylase, the enzyme involved in the production of dopamine in the brain.[3]

Plants were exploited as an alternate source for the isolation of L-DOPA and in a screening survey, more than 1000 species in 135 plant families have been screened.[4] Among them, genus *Mucuna* ( Leguminosae ) was found to contain the maximum level of L-DOPA which was successfully exploited commercially. Among the various species of *Mucuna*, *M. holtonii* (6.4% of dry weight) and *M. pruriens* (5.21% of dry weight ) were promising L-DOPA content in their seeds.[4,5]

*To whom correspondence should be addressed. E-mail: jpjbiochem@gmail.com

In naturally occurring L-DOPA synthesis, tyrosinase or tyrosine hydroxylase (EC 1.14.18.1) is the key enzyme which is a copper containing oxygen oxidoreductase and its deficiency causes oculocutaneus albinism in humans. Tyrosinase catalyzes the first two steps of melanin biosynthesis pathway, namely, the conversion of L-tyrosine into L-DOPA and further conversion of L-DOPA into dopaquinone.[6]

As the demand for L-DOPA is constantly increasing, it is a need of time to search several efficient sources of this drug. The present work embodies the seeds of *M. monosperma* as promising source of L-DOPA.

The plant *M. monosperma* is a climbing shrub producing edible seeds enclosed in a fruit pod which are restorative and possess numerous medicinal properties.[7–9] In the present work, *M. monosperma* intact soaked seeds have been used for the extraction of L-DOPA, additionally; we have studied its synthesis as well as its enhancement by adding various activators. Although we have used both intact seeds and seed powder, soaked intact seeds showed increased L-DOPA content because, soaking of the seeds in distilled water with elicitors leads to the activation of enzymes responsible for the synthesis of L-DOPA. Hence, we preferred use of intact seeds instead of the seed powder.

## Results and Discussion

**Estimation of L-DOPA.** In the present communication, we have used the Arnow's simple colorimetric method for the estimation of L-DOPA from seed powder as well as from seeds.[10] The concentrations of L-DOPA were determined using a standard graph equation ($y = 8.571x+0.056$; $R^2 =$

0.998). It gives an accurate value of L-DOPA content either in the seed powder or in the soaked seeds and can be an easy alternative to sophisticated instruments like HPLC, when a slight loss of sensitivity can be compromised.

**Determination of L-DOPA Content from the Seed Powder Soaked in Distilled Water.** The L-DOPA content in the *M. monosperma* seed powder when soaked in distilled water was found to be 4.75% (dry weight). Literature survey revealed that Mohan et al.[11] had used the acidified distilled water for the extraction of L-DOPA from seed powder of *M. monosperma* showing 4.24% yield of L-DOPA. This difference could be attributed to physico-chemical as well as environmental factors. L-DOPA in the seeds is synthesized mainly for the purpose of protection from fungal and bacterial attacks. It is a well established fact that the phenolic content of plants are influenced by number of intrinsic (genus, species, cultivar) and extrinsic (agronomic, environmental, handling and storage) factors.[12] Hence, the seeds from different locations have different L-DOPA content.

**Determination of L-DOPA Content from the Seeds Soaked in Distilled Water.** According to our literature survey, it is observed that there are no reports pertaining to estimation of L-DOPA from soaked intact seeds rather than the seed powder. Hence, in this study, we have attempted to measure the content of L-DOPA in the intact seed. During the standardization procedure, it was found that use of ascorbic acid, at a concentration of 1 mg/ml was beneficial for getting optimum L-DOPA yield of 6.58% (dry weight). In the absence of ascorbic acid, browning was accompanied with low yield (4.88%). The fundamental process in enzymatic browning is the transformation of an *o*-diphenol such as L-DOPA to the corresponding *o*-quinone, which can undergo further oxidation to brown melanin pigment.[6, 13]Ascorbic acid can prevent this enzymatic browning by trapping *o*- dopaquinone intermediate and converting it into dopahydroquinone which in turn gets converted into L-DOPA.[6] Hence, in the presence of ascorbic acid yield of L-DOPA was improved markedly.

**Effect of pH on L-DOPA Content.** Maximum yield of L-DOPA was obtained at pH 7.0 (6.58% dry weight) and pH 4.0 (5.48% dry weight) in soaked seeds and seed powder respectively (Fig. 1a and 1b). In case of seed powder and soaked seeds, pH is the critical factor, directly affecting the yield of L-DOPA. The maximum yield was observed at pH 7.0 which is contradictory to the results obtained in case of the seed powder (pH 4.0). The L-DOPA is more soluble at the acidic pH; hence, maximum L-DOPA was obtained at pH 4.0 due to its higher solubility. L-DOPA is a substrate of an enzyme polyphenol oxidase. In its active form, this enzyme transforms L-DOPA to quinones which is having maximum activity at acidic pH[13]. In case of the seed powder, polyphenol oxidase has no activity since cell integrity is lost due to the grinding which was not the case in seeds. At acidic pH, this enzyme transforms L-DOPA to DOPA quinone which leads to the decreased yield. Though the solubility of L-DOPA was relatively less at neutral pH, rate of further transformation of L-DOPA was negligible. As a result, maximum yield was obtained at pH 7.0 rather than pH 4.0 from the intact soaked seed.

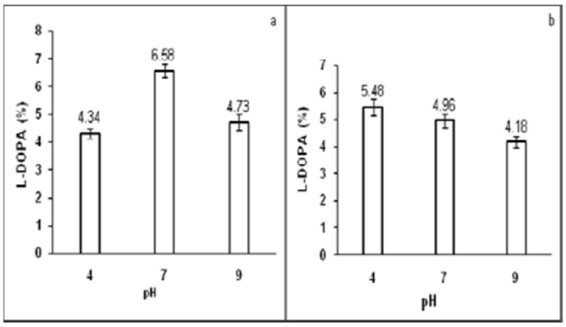

**Fig. 1. a:** Effect of pH on L-DOPA content of the intact seed
**b:** Effect of pH on L-DOPA content of the seed powder

**Effect of Germination on L-DOPA.** It was noticed that L-DOPA concentration in the seed was decreased from 6.58% (dry weight) to 2.02% (dry weight) after seed germination. It is well known that L-DOPA is an active allelochemical which inhibits other plant's growth and development.[14] According to Huizing et al.[15] callus cultures of *M. pruriens* contain 0.2% of L-DOPA in reference to cell dry weight, which can be increased only up to 1.8% by manipulating growth medium. Therefore, though the effect of L-DOPA on self plant is not known, an assumption can be made that higher concentrations might be inhibitory to cell growth, as the L- DOPA content in growing tissue like callus, and seedling is much lesser than the seed. Thus decrease in L-DOPA content might be facilitating the active cell growth.

**Effect of Incubation Period on L-DOPA.** When the effect of incubation period on L-DOPA content was observed from 12 h to 60 h, it was found that L-DOPA content of seed was found to increase from 12 h up to 48h and then decreased gradually (supplementary Fig. 1). When the seed was soaked, its biochemical pathways and enzymes were activated. During first 48 h, the tyrosine present in the seed was converted to L-DOPA and was increased till 48h. After 48 h, L-DOPA which was produced might be converted to further metabolite resulting into decreased yield.

**Effect of Elicitors on L-DOPA.** In order to boost the yield of L-DOPA several elicitors were used for enhancing the tyrosinase activity. When various concentrations of $CuSO_4$ were added as elicitors, maximum yield of L-DOPA, 9.72% (dry weight) was observed at 20 mg/dl (Fig. 2a) and in case of $CaCl_2$ maximum yield of 8.62% (dry weight) (Fig. 2b) was obtained at 10 mg/dl. Urea was better as an elicitor at the concentration of 30 mg/dl giving 10.90% (dry weight) (Fig. 2c) yield, whereas, SDS concentration was found to be optimum at 5 mg/dl which yields 11.80% (dry weight) (Fig. 2d).

Being a copper containing enzyme, the lower concentration of $CuSO_4$ improved the tyrosinase activity which in turn yielded higher L-DOPA synthesis. Calcium calmodulin mediated signaling is present in plants. So the basis behind the increase in L-DOPA content can be attributed to the increased concentration of calcium, which activates calcium binding proteins such as calmodulin or calcium dependant protein

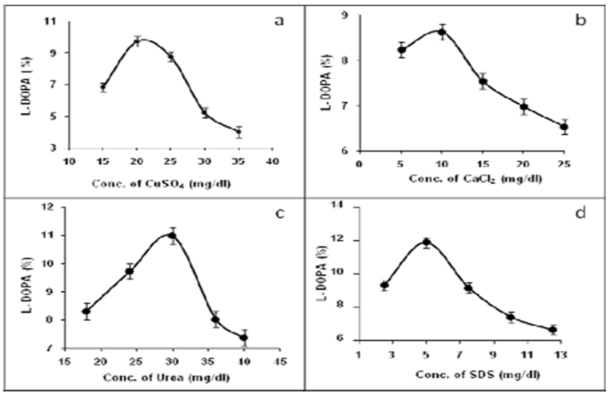

**Fig. 2.** Effect of different elicitors on L-DOPA content of the intact soaked seeds
**a:** Copper sulphate; **b:** Calcium chloride; **c:** Urea; **d:** SDS

kinases that in turn activate the other protein kinases and transcription factors.[16] These protein kinases activate tyrosinase by phosphorylating serine residues in its cytoplasmic domain.[17,18] Urea and SDS are the denaturating agents which stimulate tyrosinase activity at a lower concentration. It is suggested that the activation process involves a limited conformational changes such as a swelling of the protein rather than a dissociation or aggregation.[19] However, at higher concentrations, enzyme gets denatured and activity drops eventually. Thus all elicitors used in this study, lead to increase in the synthesis of L-DOPA through increased activity of tyrosinase. To confirm this, enzyme assay of tyrosinase with these elicitors was also performed.

**Tyrosinase Activity.** Results are shown in Table 1. Enzyme activity was lower in case of $CaCl_2$ than control perhaps calcium does not activate free enzyme directly by attaching to it but it does so by modulating synthesis of protein kinases.

**Table 1. Effect of activators on tyrosinase activity**

| Activator | Enzyme activity |
|---|---|
| Control | 0.049 ± 0.0005 |
| $CuSO_4$ | 0.072 ± 0.0011 |
| $CaCl_2$ | 0.045 ± 0.0008 |
| SDS | 0.087 ± 0.0011 |
| Urea | 0.064 ± 0.0014 |

Activities are expressed as U where one unit is mg of L-DOPA formed $min^{-1}$ mg of $protein^{-1}$.

It is clear from our observations that, the use of intact seeds is more beneficial than the seed powder. In the intact seeds, when the seeds were soaked, the biochemical environment of the cell was well maintained and soaking of the seeds in distilled water containing elicitors have activated the different enzymes present inside the cells. However, dried seed powder does not have much enzyme activity, since cell integrity was lost due to the mechanical shearing force of grinding. Hence, conversion of L-Tyrosine to L-DOPA is limited. Therefore, the yield of L-DOPA will be lower in the seed powder. There is also a possibility that the phenolic compounds secreted after seed breaking might have interfered with the L-DOPA synthesis.

**Analysis of L-DOPA Using HPTLC and HPLC.** Presence of L-DOPA in the seed powder and soaked seed extracts was confirmed by comparing with HPTLC and HPLC profile of the standard L-DOPA. In HPTLC, standard showed major peak of Rf value 0.46 corresponding to L-DOPA. Whereas, the seed powder and soaked seed extracts showed peaks at 0.46 and 0.45 respectively (Fig. 3a). The Rf values of seed powder supernatant and intact seed extract were similar to that of the standard L-DOPA. So from the Rf values and the three dimensional profile of all the three samples it was clear that both the test samples contained L-DOPA.

The HPLC elution profile of standard L-DOPA showed peak at the retention time 2.711 minutes, whereas, that of the seed powder and intact seed were at retention time 2.710 and 2.711 minutes respectively (Fig. 3b). Thus HPLC analysis confirmed the presence of L-DOPA in seed powder and soaked seeds.

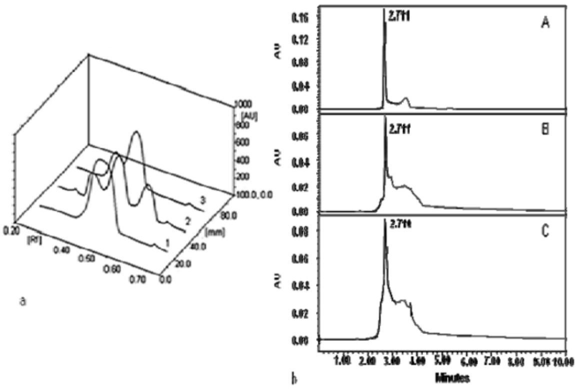

**Fig. 3.** Analysis of L-DOPA using HPTLC and HPLC
**a:** HPTLC analysis of standard L-DOPA, seed extract and seed powder extract
1: HPTLC profile of standard L-DOPA
2: HPTLC profile of soaked seed extract
3: HPTLC profile of seed powder
**b:** HPLC analysis of standard L-DOPA, seed extract and seed powder extract
A: HPLC profile of standard L-DOPA
B: HPLC profile of soaked seed extract
C: HPLC profile of seed powder

**NMR Analysis of L-DOPA.** According to our knowledge, this is the first report in which the solvent precipitation has been used to remove protein impurities from the L-DOPA present in extract of soaked seeds of *M. monosperma*. DEAE-cellulose an anion exchange elution profile of L-DOPA is shown in supplementary Fig. 2. The L-DOPA was eluted at 0.8 M NaCl. Presence of L-DOPA in the *M. monosperma* seeds was confirmed as the $^1$H NMR and $^{13}$C NMR spectrum

**Table 2. Characteristic shifts observed in the $^{13}C$ NMR spectrum.**

| Sample | Carbony | Diphenolic phenoxy | Aromatic and Indolic | Aliphatic |
|---|---|---|---|---|
| **Std L-DOPA** | 173.93 | 144.17, 143.31 | 127.51, 121.74, 116.88, 116.46 | 55.93, 35.53 |
| **Partially purified L-DOPA** | 173.94 | 144.18, 143.32 | 127.51, 121.74, 116.88, 116.46 | 55.93, 35.53 |

of test sample was identical with that of standard L-DOPA. The $^{1}H$ NMR (300 MHz, $D_2O$) showed pattern as $\delta$ 2.87 (1H, dd), $\delta$ 3.04 (1H, dd), $\delta$ 3.78 (1H, m), $\delta$ 6.58–6.78 (3H, m). Characteristic shifts observed in the $^{13}C$ NMR spectrum are shown in Table 2. $^{13}C$ NMR spectrum and $^{1}H$ NMR spectrum is shown in Fig. 4.

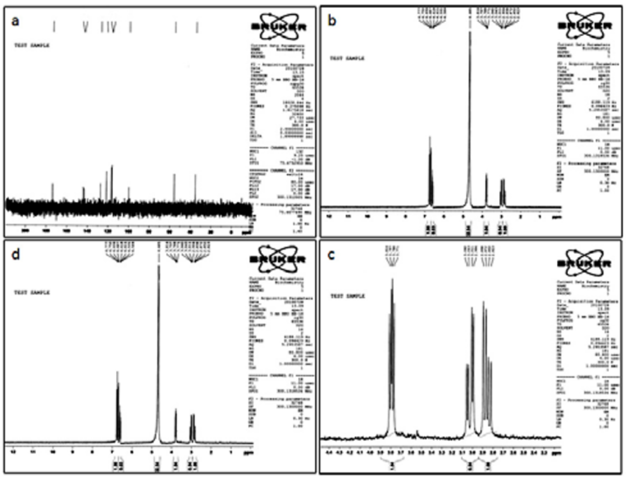

**Fig. 4.** $^{13}C$ NMR and $^{1}H$ NMR Spectrum of the isolated L-DOPA
**a.** $^{13}C$ NMR spectrum; **b–d.** $^{1}H$ NMR Spectrum

## Experimental Section

**Plant Material.** Seeds of *M. monosperma* were collected from Talsande, Kolhapur district, Maharashtra, India. The herbarium accession number of the plant is MMS 2273. 'The specimen was deposited at Department of Botany, Shivaji University, Kolhapur, India.

**Determination of L-DOPA Content from the Seed Powder Soaked in Distilled Water.** Seeds of *M. monosperma* were grounded to a fine powder and 1 g powder was soaked in 200 ml distilled water for 48 h at 37 ± 2°C. This mixture was then centrifuged at 11,739 g for 20 min. Supernatant was collected and filtered through Whatman filter paper and 1 ml of the filtrate was subjected for the estimation of L-DOPA by the Arnow's[10] method.

**Determination of L-DOPA Content from the Seeds Soaked in the Distilled Water.** After surface sterilization with absolute alcohol; seed coats from dried seeds of *M. monosperma* were removed and seed dry weight was measured. Then 10 (± 0.2) g seeds (3 seeds) were soaked in 300 ml sterile distilled water. After 48 h, seeds were harvested and crushed in mixer with 150 ml 20 mM sodium phosphate buffer (pH 7.0) containing 1mg/ml ascorbic acid. Supernatant was collected by applying centrifugal force of 11,739 g for 20 min. Remaining residue was subjected for the same procedure, so as to recollect remaining L-DOPA. The supernatants obtained from these two steps were used for the estimation of L-DOPA. The distilled water, in which the seeds were soaked, was also estimated for extracellular L-DOPA content.

**Effect of pH on L-DOPA Content.** Effect of different pH on the content of L-DOPA was studied by using buffers having pH 4, 7 and 9. 20 mM Acetate buffer (pH 4.0), 20 mM phosphate buffer (pH 7.0) and 20 mM Tris-HCl buffer (pH 9.0) were used for the soaking of seeds and seed powder for 48 h at 37±2 °C. Soaked seeds and seed powder were then subjected for estimation of L-DOPA as described in earlier sections.

**Effect of Germination on L-DOPA Content.** L-DOPA content in the seeds before and after germination was monitored. Seeds were surface sterilized and allowed to germinate aseptically.

**Effect of Incubation Period on L-DOPA Content.** Seeds were soaked in 20 mM phosphate buffer (pH 7.0) for different time intervals like 12 h, 24 h, 36 h, 48 h, 60 h and L-DOPA content was determined to elucidate the effect of incubation period on L-DOPA content of the seed.

**Effect of Elicitors on L-DOPA Content.** Elicitors like, copper sulphate ($CuSO_4$), calcium chloride ($CaCl_2$), urea and sodium dodecyl sulphate (SDS) were used to enhance the production of L-DOPA. The seeds were soaked in different concentrations of above mentioned elicitors for 48 h and the L-DOPA content was estimated.

**Tyrosinase Assay.** Enzyme activity from the seeds was evaluated by soaking seeds in the distilled water for 24 h. The seeds were homogenized in ice cold sodium phosphate buffer (20 mM, pH 7.0). Ascorbic acid (1 mg/ml) was added to the extraction buffer and then the mixture was centrifuged at 11,739 g for 20 min. The resulting supernatant was used as a source of crude enzyme. Tyrosinase activity was determined by the method as described below. The final reaction mixture contained 5 mM L-tyrosine, 50 mM potassium phosphate buffer (pH 7.0) and 0.2 ml enzyme. The reaction mixture was incubated at 37°C for 30 min. The L-DOPA produced in the assay was measured by the Arnow's method.[10] One unit of tyrosinase activity is defined as mg of L-DOPA produced mg of protein$^{-1}$min$^{-1}$. Protein content was determined by Lowry method. Enzyme activity in the presence of elicitors was also studied. For the activator study, reaction mixture was supplemented with 0.1 ml activators namely $CuSO_4$ (0.2 mg/ml), $CaCl_2$ (0.1 mg/ml), SDS (0.05 mg/ml) and urea (0.3 mg/ml).

**Analysis of L-DOPA Using HPTLC and HPLC.** High performance thin layer chromatography (HPTLC) and High performance liquid chromatography (HPLC) analysis was performed by using the method described by Surwase et al.[20]

**NMR Analysis of L-DOPA.** Purified L-DOPA was used for the NMR analysis. The isolation of L-DOPA was performed from the seeds by the method described earlier and used further for purification. To make the L-DOPA free from protein impurities, the supernatant obtained after centrifugation was subjected to cold acetone precipitation and centrifuged at 11,739 g for 20 min. The supernatant was concentrated to one tenth of its volume under vacuum in a rotary evaporator and stored at 4°C for 24 h. The precipitate formed was then recovered by filtration and dissolved in phosphate buffer (20 mM, pH 7.0) which was loaded on DEAE-cellulose, an anion exchange column (cylindrical glass column with 30cm height and 1 cm diameter) equilibrated with the same buffer at a flow rate of 1ml/min. The column was washed with the same buffer (two times of the column volume) and then retained L-DOPA was eluted with a linear NaCl gradient from 0 to 1.0 M. Fractions containing L-DOPA were then pooled together, evaporated to obtain powder and subjected for NMR analysis. The $^{1}H$ NMR and $^{13}C$ NMR of purified L-DOPA was carried out using Bruker Ac 200 at 24°C in deuterium oxide.

**Statistical Analysis.** All the experiments are performed in triplicate and analyzed by using Microsoft excel and Graph pad Instat 3 software.

## Conclusion

The present work demonstrates that *M. monosperma* is an efficient natural source of L-DOPA and a good alternative to *M. pruriens* which is a current source of L-DOPA. Extensive harvesting of *M. pruriens* for the supply of L-DOPA, would pose a danger of its depletion from the natural wealth, hence, *M. monosperma*, the plant under study, will be an added source for the isolation of L-DOPA. Interestingly, soaked seeds of *M. monosperma* showed maximum yield of L-DOPA than *M. pruriens* or any other plant earlier reported. This study also underlines the use of soaked seeds rather than seed powder, as it allowed manipulation of biochemical reactions to achieve higher production from a limited source.

## Electronic Supplementary Material

Supplementary material is available in the online version of this article at http://dx.doi.org/ 10.1007/s13659-011-0051-3 and is accessible for authorized users.

## Acknowledgments

We thank Mr. U. B. Jagtap for the supply of seed material. Mr. S. A. Inamdar thanks UGC, New Delhi for BSR meritorious fellowship. Prof. V. A. Bapat wishes to thank CSIR, New Delhi for Emeritus scientist fellowship. Ms. S. M. Joshi thanks DBT, New Delhi for the grant of SRF. The authors like to acknowledge Dr. D. H. Dagade and Dr. U. V. Desai, Department of Chemistry, Shivaji University for NMR analysis of L-DOPA.

**Open Access** This article is distributed under the terms of the Creative Commons Attribution License which permits any use, distribution, and reproduction in any medium, provided the original author(s) and source are credited.

## Electronic supplementary material

Supplementary material, approximately 87.5 KB.
