# Supplementary material for: Innovative use of intact seeds of Mucuna monosperma Wight for improved yield of L-DOPA
Source: Nat Prod Bioprospect. 2012 Feb 7;2(1):16–20. doi: 10.1007/s13659-011-0051-3 (PMC4131573; doi:10.1007/s13659-011-0051-3)

Electronic Supplementary Material

Innovative use of intact seeds of *Mucuna monosperma* Wight for improved yield of L-DOPA

Shrirang INAMDAR, Swati JOSHI, Jyoti JADHAV,* and Vishwas BAPAT

Department of Biotechnology, Shivaji University, Kolhapur 416004, India

Received 31 December 2011; Accepted 1 February 2012

Table of contents

Figure 1: Effect of incubation period on L- DOPA content of the intact seed

Figure 2: Elution profile of L- DOPA from DEAE cellulose column

—◆—Concentration of L- DOPA (mg mL^{-1})

*To whom correspondence should be addressed. E-mail: jpjbiochem@gmail.com

Supplementary Fig. 1.

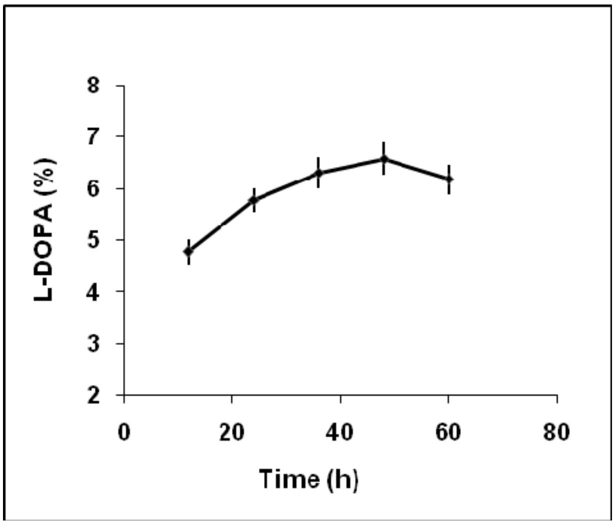

Supplementary Fig. 2.

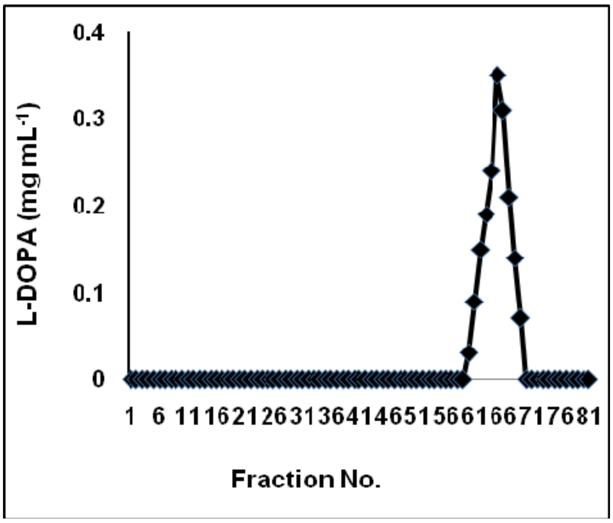

Supplement: Supplementary file 1 — Supplementary material, approximately 87.5 KB. [file 13659_2011_51_MOESM1_ESM.pdf]
